# Minimally invasive thoracoscopic resection of a micronodular thymoma with lymphoid stroma via a subxiphoid single-incision approach: A case report

**DOI:** 10.1097/MD.0000000000039637

**Published:** 2024-09-06

**Authors:** Qiang Wu, Kun Qiao, Xiaoming Zhang, Zizi Zhou

**Affiliations:** a Department of Cardio-Thoracic Surgery, Shenzhen University General Hospital, Shenzhen, China; b Department of Thoracic Surgery, National Clinical Research Center for Infectious Disease, Shenzhen Third People’s Hospital, Shenzhen, China; c The Second Affiliated Hospital, School of Medicine, Southern University of Science and Technology, Shenzhen, China.

**Keywords:** case report, lymphoid stroma, micronodular thymoma, subxiphoid single-incision approach, thoracoscopic resection

## Abstract

**Rationale:**

This study aims to present a novel surgical approach for the resection of anterior mediastinal tumors, specifically focusing on micronodular thymoma with lymphoid stroma (MNT), a rare and distinct variant of thymoma. The single subxiphoid incision technique, although reported in limited cases, offers a minimally invasive option with potential benefits. We report the case of a 76-year-old male who underwent this innovative procedure and was diagnosed with MNT, providing insight into the management and outcomes of this rare pathology.

**Patient concerns:**

The patient presented for the excision of an anterior mediastinal tumor, with the surgery facilitated by sternal hooks to improve visualization. The rarity of MNT and its unclear prognosis underscore the need for enhanced diagnostic accuracy and tailored treatment strategies.

**Diagnoses:**

Initially diagnosed preoperatively with a thymic cyst, the patient’s final diagnosis was revised to MNT following surgery, highlighting the diagnostic challenges associated with this rare tumor.

**Interventions:**

The tumor was successfully removed using minimally invasive thoracoscopic surgery through a subxiphoid single-incision, demonstrating the feasibility and potential advantages of this approach.

**Outcomes:**

The patient had a favorable postoperative course, with a swift recovery and no complications, and remained in good health without signs of relapse at the 9-month follow-up.

**Lessons:**

This case underscores the importance of recognizing the unique pathological features of MNT and the need for a cautious diagnostic approach to differentiate it from other cystic lesions. Additionally, the successful use of single-port thoracoscopy under the xiphoid process for the removal of thymic tumors suggests its potential as an effective surgical method for these challenging cases.

## 1. Introduction

Micronodular thymoma (MNT) with lymphoid stroma, also called micronodular thymoma with lymphoid B-cell proliferation, is an extremely rare and unique subtype of thymic epithelial tumor, accounting for <5% of all thymomas.^[1,2]^ In 1999, Suster and Moran originally reported it as a thymic neoplasm characterized by multiple small tumor nodules that are separated by abundant B lymphocytes with prominent germinal centers.^[3]^ Subsequently, it was found that MNT also contains a considerable number of mature T cells and the majority show lymphoid follicular hyperplasia, with the T cells playing an important role in the immune response.^[4]^ Because of the extremely small number of reported cases, there is limited understanding of the pathogenesis-mediated lymphoid hyperplasia and prognosis of MNT. In recent years, with the advancements and popularization of minimally invasive technology, thoracoscopic methods are being widely used. We have also reported our remarkable achievements in thoracoscopic surgery using the subxiphoid approach.^[5]^ A single subxiphoid incision is a novel approach for perform thoracoscopic surgery, which has been less frequently employed for anterior mediastinum tumors. As MNT is a rare variant of thymoma, there have been few case reports on MNT. Here, we report a rare case of MNT with lymphoid stroma that was resected using the thoracoscopic approach via a subxiphoid single incision. This novel technique has not been previously reported for resection of an anterior mediastinal tumor.

## 2. Case presentation

**Figure 1.**
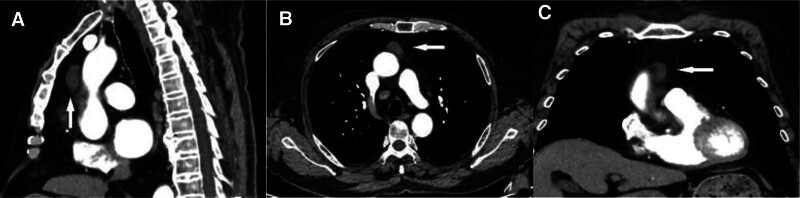
(A–C) Chest computed tomography shows a well-defined lesion in the anterior mediastinum (arrow), which exhibits no significant enhancement of its cystic component.

**Figure 2.**
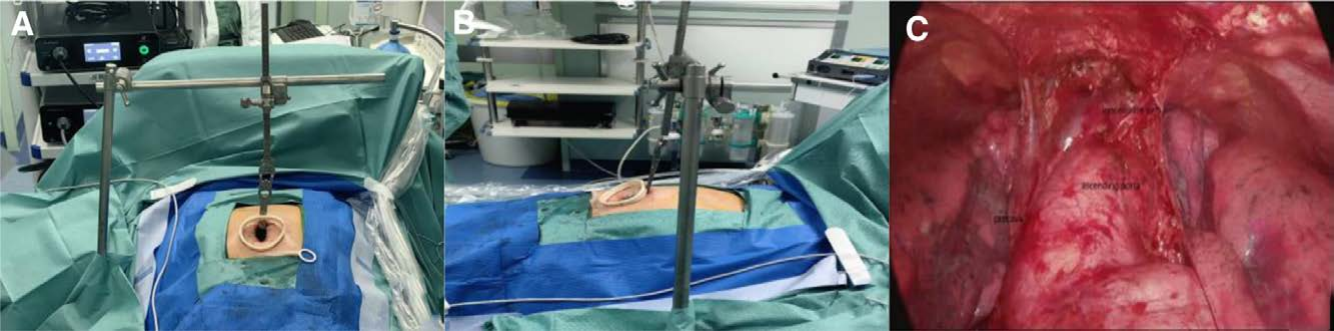
(A and B) Location of the single subxiphoid incision and application of the sternal retractor. (C) View after anterior mediastinal tumor resection.

**Figure 3.**
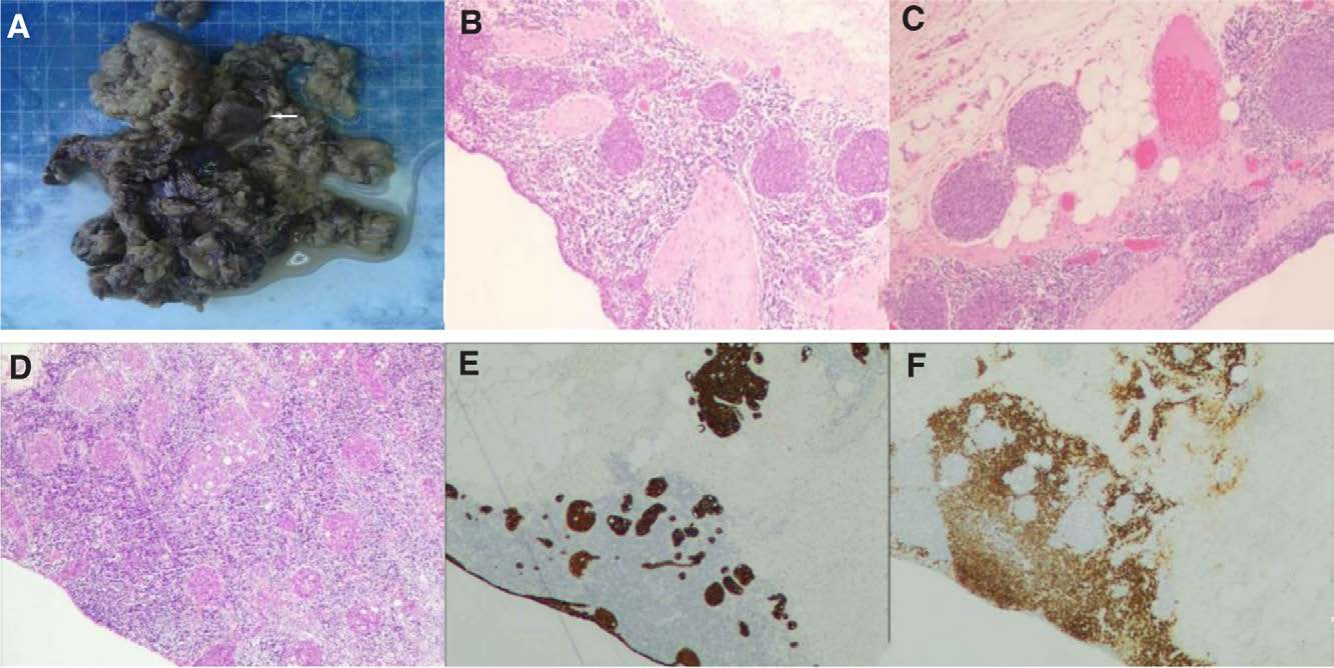
(A) Gross examination showing the resected thymoma (arrow) amidst the adipose tissue. (B–D) Microscopically, multiple tumor nodules composed of spindle epithelial cells are dispersed throughout a lymphoid-rich stroma featuring prominent germinal centers and exhibiting a cystic growth pattern. (E and F) On immunohistochemical staining, the tumor nodules were positively stained for CK19, whereas the surrounding stroma was positive for CD20.

A 76-year-old man presented to the Thoracic Surgery Department with an anterior mediastinal mass detected by chest computed tomography (CT). He had coronary heart disease and had undergone surgery for lipoma but did not have myasthenia gravis or other autoimmune diseases. There was no history of tobacco use, smoking, or drinking alcohol, and no significant family history. No abnormalities were observed on physical or laboratory examination. Chest CT revealed an abnormally dense shadow of 27 mm  × 14 mm size in the anterior mediastinum, which had clear boundaries and did not show significant enhancement, most likely a thymic cyst (Fig. 1A–C). Subsequently, the patient underwent minimally invasive thoracoscopic surgery via a single subxiphoid incision to remove the anterior mediastinal tumor using a special instrument, the sternal hook (Fig. 2A and B). No pleural adhesion or invasion of the surrounding structures was observed during surgery, and a nodule-shaped tumor, along with the peripheral adipose tissue was removed (Fig. 2C). The gross and microscopic features of the lesion are shown in (Fig. 3A–F). The patient was diagnosed with MNT with cystic lesions. The patient recovered satisfactorily and was discharged without any complications. He remained well and did not relapse during 9 months of follow-up.

## 3. Discussion

MNT is an unusual variant of primary thymic neoplasms which has unique histological features and immune phenotype. It is characterized by numerous nodules formed by neoplastic epithelial cells, abundant lymphoid stroma, and germinal centers. In the 2015 World Health Organization (WHO) classification, it was listed as an independent type, owing to the histological pattern.^[6]^ This tumor is more common in middle-aged and older people, usually those over 40 years of age, with no significant sex differences.^[7]^ The tumor is mostly found in the anterior mediastinum, and there are few reports of ectopic MNT in the neck or suprasternal fossa, which may be related to the origin of the thymus.^[8]^ Most patients are asymptomatic, but a few may experience chest tightness or pain. Apart from being accompanied by other types of thymomas, there is generally no paraneoplastic syndrome, such as myasthenia gravis. Most MNTs are discovered incidentally during radiographic examination. Cystic changes are common in MNT, and almost half of the patients have cystic lesions on imaging.^[9]^ It is sometimes difficult to distinguish thymomas from cystic lesions and thymic cyst.^[10]^ A study analyzed the imaging features of contrast-enhanced CT in MNT, and found that they were mostly cystic-solid with multiple cystic and enhanced solid components, with no invasive or malignant signs. These findings can help in diagnosing MNT.^[11]^ Microscopically, MNTs have unique histological features, including small nodules composed of tumor epithelial cells separated by abundant lymphoid stroma. Within the lymphoid stroma, follicles with distinct germinal centers are found and plasma cells infiltrate in varying numbers. Epithelial cells are spindle-shaped or oval-shaped, with no obvious cell abnormalities, and usually without Hassall corpuscles or perivascular spaces.^[12]^ Immunohistochemically, some epithelial markers, such as CK pan, AE1/3, CK5/6, CK19, CK8/18, and CD20 are often expressed by tumor epithelial cells. Most the lymphoid stroma and B lymphocytes were positive for CD2, with a small number of T lymphocytes interspersed in between.^[13]^ It is not difficult to diagnose MNT based on histopathology and immunohistochemistry.

The potential pathogenesis of MNT with interstitial lymphoid hyperplasia is still obscure. Overgrowth of the lymphoid stroma, which divides sheets of tumor cells into small islands, may be caused by the host immune response to tumor antigens.^[14]^ This response pattern is commonly observed throughout the body during various neoplastic processes.^[15]^ Regarding the reason for the immunohistochemical pattern, Ishikawa found that the Langerhans cells in epithelial nodules carry the tumor antigens and migrate to the lymphoid stroma, where they mature into dendritic cells and cluster with CD4-positive helper T lymphocytes, thus activating the lymphocytes through a series of antigen stimuli and leading to the formation of lymphoid follicles. Langerhans cells play an important role in the pathogenesis of mesenchymal lymphoid tissue proliferation in MNT.^[16]^ It has been postulated that MNT may provide a high-risk environment for the occurrence of mucosal-associated lymphoid tissue lymphoma. Strobel reported that high levels of chemokines are expressed in the neoplastic epithelial cells of MNT, which can promote lymphocyte recruitment into the stroma of MNT and induce B-cell monoclonization, leading to the development of mediastinal lymphoma.^[17]^ Furthermore, Zaitsu reported a case of micronodular thymic carcinoma arising from an MNT.^[18]^

The prognosis of MNT remains unclear because of its rarity.^[19]^ It is currently thought to be a slow-growing borderline neoplasm with a well-defined capsule or only focal microinvasion of the peri-thymic fat. Few cases of recurrence after surgery and no cases of metastasis or tumor-related deaths have been reported. Relevant studies have shown that patients with solid cystic tumors have a better prognosis, whereas patients with solid tumors and capsule invasion have a higher risk of recurrence. Kaminuma reported a case of recurrent MNT as a result of incomplete surgical resection of the tumor 10 years ago. After complete resection of the recurrent tumor, the patient had a favorable prognosis, with no recurrence or distant metastasis were reported.^[20]^

Subxiphoid thoracoscopic surgery is a new surgical technique developed in recent years, which has achieved good results in the treatment of anterior mediastinal tumors.^[21]^ Previous studies have described thymectomy and lung wedge resection using a subxiphoid surgical approach.^[22]^ Compared to unilateral thoracoscopic surgery, it can provide better operative views of the anterior mediastinum that are close to those provided by median sternotomy, clearly exposing the bilateral phrenic nerves and superior horn of the thymus and reducing the possibility of intraoperative injury. Thus, it has outstanding advantages in the surgical treatment of anterior mediastinal tumors. It avoids damage to the intercostal nerve and significantly reduces postoperative pain. Additionally, less trauma and fewer complications lead to faster patient recovery. The incision position is low, which leads to better cosmetic results.^[23,24]^ However, owing to the narrow space in the anterior mediastinum, it is difficult to master the operation via the subxiphoid approach.^[25]^ In the present case, we excised the anterior mediastinal tumor through thoracoscopic surgery via a subxiphoid single incision using sternal hooks. Elevating the sternum significantly broadens the space behind it, facilitating removal of the tumor and thorough dissection of the fat tissue around the anterior mediastinum, while avoiding residual tumor. Furthermore, it is easy to master, shortens the operation time, and does not increase complications. In our institute, tumors <5 cm in diameter or with no invasion of phrenic nerves, pericardium, or major vessels were treated through minimally invasive thoracoscopic surgery via a subxiphoid single-incision with the help of sternal hooks; otherwise, open approaches such as median sternotomy were performed.^[26]^

In summary, MNT is a very rare subtype of thymoma with unique pathological features and a favorable prognosis. It has biological behaviors different from those of other types of thymoma. Due to its rarity, its diagnosis poses certain challenges. Therefore, it is crucial to identify the unique pathological characteristics of this rare disease. Even in cases with strong suspicion of a thymic cyst, clinicians should differentiate MNT from cystic lesions to ensure that the patients receive appropriate treatment.^[27]^ In this case, we used special instruments, such as sternal hooks, to perform single-port thoracoscopy under the xiphoid process to completely remove the thymic tumor, which has a certain clinical reference value.

## Author contributions

**Conceptualization:** Qiang Wu, Kun Qiao, Zizi Zhou.

**Data curation:** Qiang Wu, Kun Qiao, Zizi Zhou.

**Formal analysis:** Qiang Wu, Kun Qiao, Zizi Zhou.

**Funding acquisition:** Zizi Zhou.

**Methodology:** Qiang Wu, Kun Qiao, Zizi Zhou.

**Supervision:** Xiaoming Zhang.

**Writing – original draft:** Qiang Wu, Zizi Zhou.

**Writing – review & editing:** Xiaoming Zhang, Zizi Zhou.
